# A novel closed reduction with extension block and flexion block using Kirschner wires and microscrew fixation for mallet fractures

**DOI:** 10.1007/s00776-013-0526-7

**Published:** 2014-01-23

**Authors:** Haruhiko Shimura, Yoshiaki Wakabayashi, Akimoto Nimura

**Affiliations:** 1 Department of Orthopaedic Surgery, National Printing Bureau Tokyo Hospital, 2-3-6 Nishigahara, Kita-ku, Tokyo, 114-0024 Japan; 2 Department of Orthopaedic and Spinal Surgery, Graduate School of Medical and Dental Sciences, Tokyo Medical and Dental University, 1-5-45 Yushima, Bunkyo-ku, Tokyo, 113-8519 Japan; 3 Department of Clinical Anatomy, Graduate School of Medical and Dental Sciences, Tokyo Medical and Dental University, 1-5-45 Yushima, Bunkyo-ku, Tokyo, 113-8519 Japan

## Abstract

**Background:**

Some patients with mallet fractures who undergo extension block pinning complain of exposed wires, which delay their return to sports and causes inconvenience while performing tasks that require the use of hands during the early postoperative period. The purpose of this retrospective study was to present and evaluate a novel surgical procedure for mallet fractures.

**Methods:**

We treated 20 patients (14 males and six females; mean age, 38.4 years; range 17–68 years) with displaced mallet fractures involving >30 % of the articular surface using the closed reduction and microscrew fixation between January 2009 and January 2012. The distal interphalangeal joint (DIP) joint was immobilized with a splint for 1–3 weeks on an individual case basis. According to Wehbe and Schneider’s classification, there were 12 type IB, six type IIB, and two type IA fractures. The mean follow-up duration was 12.6 months (range 6–31 months).

**Results:**

Bone union was achieved in all patients within a mean period of 6.8 weeks, with no incidence of infection, skin necrosis, permanent nail deformity, or secondary osteoarthritis. Only two complications—temporary nail ridging in one patient and a dorsal bump caused by the screw in one patient—were observed. Minimum postoperative displacement was observed in one patient, for whom immobilization with a splint was continued for 4 weeks. Articular incongruity was <1.0 mm in four patients and 1.0–2.0 mm in two patients. Mean DIP joint extension loss was 6.5° and mean flexion was 67.8°. The surgical outcomes were excellent in seven patients, good in nine, and fair in four according to Crawford’s evaluation criteria.

**Conclusion:**

Our novel surgical procedure combining closed reduction with extension block and flexion block using Kirschner wires and microscrew fixation produces good clinical results with relatively few complications.

## Introduction

Although mallet fracture is a common sports or work-related injury, its treatment remains controversial. Wehbe and Schneider concluded that splinting is a safe and reliable treatment for most mallet injuries and that reduction is not crucial because of bone remodeling [1]. However, surgical treatment has been suggested for fractures involving >30 % of the articular surface or for fractures with volar subluxation [2, 3]. Patients with such fractures are at an increased risk for secondary osteoarthritis and esthetically unacceptable outcomes. Various surgical techniques such as Kirschner wire (K-wire) pinning [4–6], pull-out wiring [7], compression pin fixation [8], hook plate fixation [9], and microscrew fixation [10] have been described. Although open reduction has been proposed, soft tissue damage may occur with this approach; furthermore, complications such as infection, skin necrosis, nail deformities, and joint stiffness are a possibility [11]. Therefore, closed reduction with extension block was described in 1988 by Ishiguro et al. [5] to avoid the disadvantages of open reduction. As per previous studies, this surgical procedure is less invasive and provides stable postoperative results [4–6]. However, many patients complain about exposed wires, which delay their return to sports and causes inconvenience while performing tasks requiring the hands during the early postoperative period. Therefore, we introduced a novel microscrew fixation technique that leaves no exposed wire on the skin. This report presents our novel closed reduction technique with extension block and flexion block using K-wires followed by microscrew fixation for mallet fractures and retrospectively evaluates the clinical results of this procedure.

## Patients and methods

We treated 20 displaced mallet fractures (14 males and six females; mean age 38.4 years; range 17–68 years) using our novel technique between January 2009 and January 2012. The institutional review board approved this study, and informed consent was obtained from each patient. The right hand was involved in 14 patients and the left in six. The fingers affected included seven middle fingers, five ring fingers, five little fingers, and three index fingers. Twelve injuries were sports related while eight were fall related. No patient had any medical history of bone diseases that could have influenced surgical outcomes. The indication for surgery was the presence of a displaced fracture involving >30 % of the articular surface. Preoperative lateral X-rays were used to determine fragment size. Six cases had volar subluxation of the distal phalanx while fourteen did not. According to Wehbe and Schneider’s classification [1] (Table 1), there were 12 type IB, six type IIB, and two type IA fractures. The mean duration from injury to surgery was 9.2 days (range 1–16 days). Functional outcomes were determined using Crawford’s evaluation criteria [12] (Table 2). Full flexion of the distal interphalangeal (DIP) joint was considered for the angle of the uninjured joint on the opposite hand. Radiographs were obtained at 2, 4, 6, 8, and 10 weeks, followed by every 6 months after bone union was complete. The mean follow-up duration was 12.6 months (range 6–31 months).

**Table 1:** Wehbe and Schneider’s classification [1]

Classification	Number
Type I (no joint subluxation)	
Subtype A	2
Subtype B	12
Subtype C	0
Type II (subluxation of DIP joint)	
Subtype A	0
Subtype B	6
Subtype C	0
Type III (physis of the distal phalanx involved)	
Subtype A	0
Subtype B	0
Subtype C	0

Subtype A: <1/3rd of the articular surface

Subtype B: 1/3–2/3rd of the articular surface

Subtype C: >2/3rd of the articular surface

**Table 2:** Crawford’s evaluation criteria [12]

Excellent	Full distal joint extension, full flexion, no pain
Good	0°–10° Of extension deficit with full flexion, no pain
Fair	10°–25° Of extension deficit, any flexion loss, no pain
Poor	>25° Of extension deficit, persistent pain

### Surgical technique

**Fig. 1:**
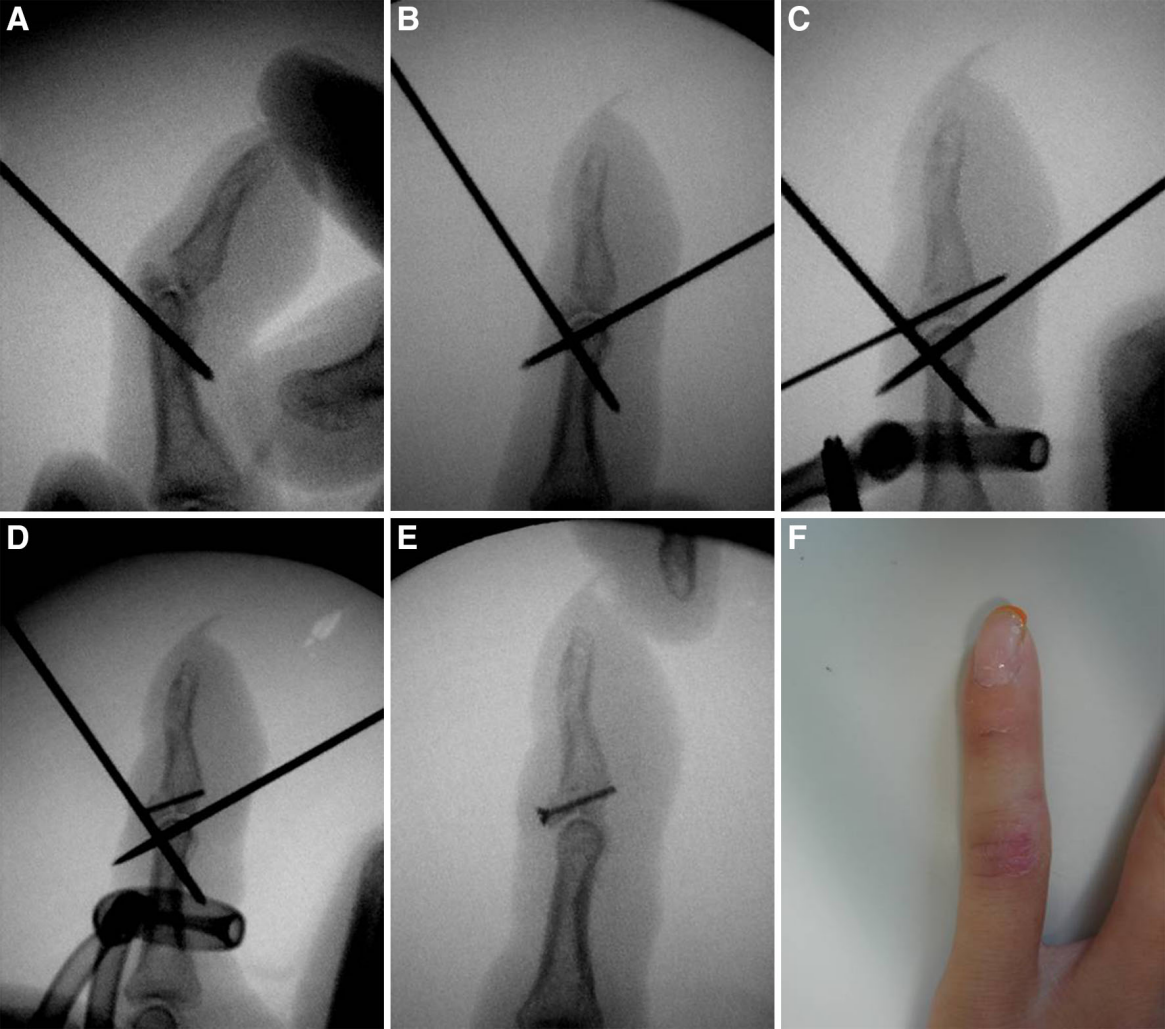
**a** The Kirschner wire for extension block is inserted. **b** The Kirschner wire for flexion block is inserted. **c** A third Kirschner wire is inserted as a substitute for the drill hole. **d** A 1.0-mm microscrew is inserted. **e** The blocking Kirschner wires are removed. **f** An image of the wound 3 days after surgery

**Fig. 2:**
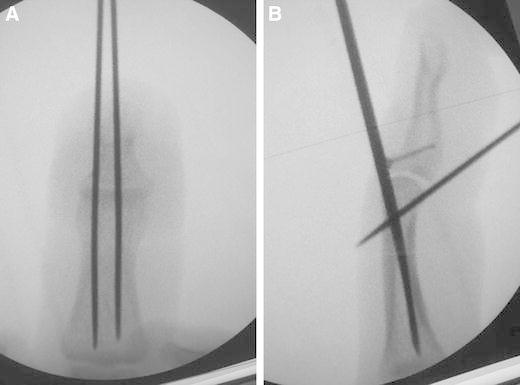
**a** Two extension blocks are performed since January 2011. **b** A 1.0-mm microscrew is inserted between the two extension blocks

This novel procedure, combining extension block and flexion block using K-wires with closed reduction followed by microscrew fixation, is a less invasive procedure and leaves no exposed wires on the skin. All procedures were performed under digital block anesthesia with an image intensifier. With the DIP joint passively maintained in maximum flexion, the first 1.0-mm K-wire was placed into the head of the middle phalanx from the dorsal side of the bone fragment for extension block (Fig. 1a). Since January 2011, we have been performing a two extension block technique to stabilize the bone fragment (Fig. 2a, b), and this technique was used in six patients in this study. The K-wire on the ulnar side was inserted 3 mm apart from and parallel to the radial side K-wire. The second 1.0-mm K-wire was inserted into the head of the middle phalanx from the center of the volar side for flexion block following closed reduction by extending the DIP joint (Fig. 1b). Closed reduction was achieved without inserting a K-wire into the bone fragment. We used the third 0.7-mm K-wire as a substitute for a 0.7-mm drill bit. This wire, which is perpendicular to the fracture line, was inserted through the center of the bone fragment from the dorsal to the volar side (Fig. 1c). The length of the screw can be determined using another K-wire of the same length and an image intensifier. A digital tourniquet with a Nelaton’s catheter tube was placed. A 1.0-mm microscrew (Nippon Martin K.K., Osaka, Japan) was inserted following placement of a transverse minimum incision (approximately 3 mm) along the skin crease (Fig. 1d). The first and second blocking K-wires were removed once the screw was fixed (Fig. 1e). The congruity and stability of the DIP joint were confirmed under the image intensifier. Suturing of the wound was not considered necessary; this was confirmed by an image obtained 3 days after surgery, which showed that the wound had already healed (Fig. 1f). The DIP joint was immobilized with a splint for 1–3 weeks on an individual case basis. Passive and active DIP joint exercises were encouraged after splint removal.

## Results

Bone union was achieved in all 20 fractures within a mean period of 6.8 weeks (range 4–10 weeks). Reduction achieved during surgery was maintained in 19 patients with no secondary displacement. Minimum displacement was observed in one patient and immobilization with a splint was continued for 4 weeks. At the final follow-up, anatomical reduction was observed in 14 patients and articular incongruity was <1.0 mm in four patients and 1.0–2.0 mm in two. Mean DIP extension loss was 6.5° (range 0°–20°) and mean DIP flexion was 67.8° (range 45°–80°). No infection, skin necrosis, permanent nail deformity, or secondary osteoarthritis was observed. Only two complications were observed; nail ridging in one patient, which disappeared after 5 months, and a dorsal bump caused by the screw in one patient, which was removed. According to Crawford's evaluation criteria, the surgical outcomes were excellent in seven patients, good in nine, and fair in four.

### Case

**Fig. 3:**
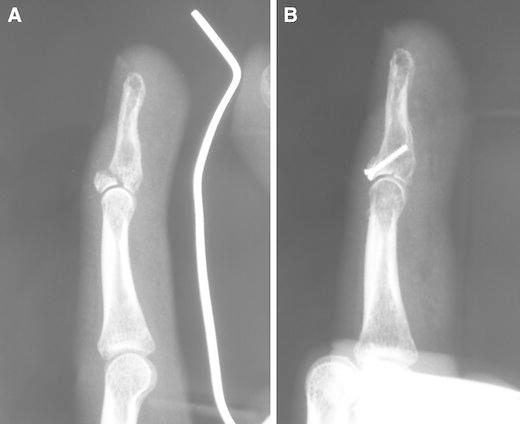
**a** Radiograph of a 38-year-old man with a type IIB mallet fracture. **b** Radiograph showing achievement of anatomical reduction

A 38-year-old male industry worker injured his left middle finger while he was on duty. Radiographs showed a displaced mallet fracture involving >50 % of the articular surface, without volar subluxation of the distal phalanx (Fig. 3a). The fracture was type IB according to Wehbe and Schneider’s classification. He wished to resume work as early as possible; therefore, we treated the fracture using the procedure described above. We used a 0.7-mm K-wire as a substitute for a 0.7-mm drill bit and inserted a 1.0-mm microscrew with closed reduction (Fig. 3b). As a result, the patient could resume work with taping 10 days after surgery. This patient exhibited active DIP motion from 0° to 75° without pain 12 months after surgery, and his surgical outcome was excellent as per Crawford’s evaluation criteria.

## Discussion

Various surgical techniques for displaced mallet fractures, such as K-wire pinning, pull-out wiring, compression pin fixation, hook plate fixation, and microscrew fixation have been described. However, the ideal treatment for fractures involving >30 % of the articular surface remains controversial. Ishiguro’s method [5] is easier and less invasive than most currently available methods; moreover, it facilitates closed reduction and is a reliable method that permits indirect anatomical reduction of the fracture. We routinely performed Ishiguro’s method at our hospital for cases of mallet fracture with involvement of >30 % of the articular surface. However, some patients complained of exposed wires that delayed their return to physical activity and affected their work during the early postoperative period. Therefore, we developed a novel microscrew fixation procedure that leaves no exposed wire, allows rapid healing with good mobility, and does not delay the patient’s return to physical activity and work.

The microscrew fixation procedure for mallet fractures was described in 2004 by Kronlage et al. [10]. In their surgical technique, the fracture site is opened and cleared of hematoma and callus, the fragment is repositioned and held in a reduced position with forceps or a towel clip, and two or more 0.8-mm microscrews are inserted. Hiwatari et al. [13] reported the chased method for mini-screw fixation in cases of mallet fractures in 2012. The chased method is a percutaneous procedure in which the fixation screw chases the K-wire as a substitute for a drill bit. We think that the microscrew fixation procedure for mallet fracture has two pitfalls. First, there is a possibility of inducing cracks in the bone fragment, and second, there can be a reduction loss during drilling and screw insertion. We consider that less invasive reduction is achieved using extension and flexion block without forceps. To decrease the possibility of cracking a bone fragment, we use a third 0.7-mm K-wire as a substitute for the 0.7-mm drill bit and insert a 1.0-mm microscrew into the bone fragment using blocked K-wires. A 1.5-mm microscrew is often inserted depending on the bone fragment size.

Lucchina et al. [14] reported a comparison among three different techniques (extension block pinning, K-wires used as joysticks and miniscrew fixation with open reduction and internal fixation) for unstable mallet fractures, and no statistically significant difference was observed in the functional results. Open reduction and screw fixation allows for earlier mobilization and faster return to work. If the treatment results of these methods are not much different, then an early return to sports or work and a low rate of complications are important. Most surgical techniques have the disadvantages of an open incision, difficulty in achieving earlier mobilization, and the incidence of nail deformity, skin necrosis, and pin tract infection. Kang et al. [11] reported that 41 % of surgically treated mallet fractures developed postoperative complications. Therefore, we consider that the complication rate in our study (2/20 patients) was acceptable.

## Conclusion

Our novel surgical procedure combining closed reduction with extension block and flexion block using K-wires followed by microscrew fixation for mallet fractures produces good clinical results with relatively few complications.
